# BPM Proteins Modulate Heat Stress Response in *Arabidopsis thaliana* Seedlings

**DOI:** 10.3390/plants14131969

**Published:** 2025-06-27

**Authors:** Sandra Vitko, Dunja Leljak-Levanić, Nataša Bauer, Željka Vidaković-Cifrek

**Affiliations:** Department of Biology, Faculty of Science, University of Zagreb, Horvatovac 102a, 10000 Zagreb, Croatia; sandra.vitko@biol.pmf.unizg.hr (S.V.); dunja.leljak-levanic@biol.pmf.unizg.hr (D.L.-L.); natasa.bauer@biol.pmf.unizg.hr (N.B.)

**Keywords:** *amiR-bpm*, BTB/POZ-MATH, *DREB2A*, *HSFA3*, HSPs, JIP-test, *oeBPM1*, oxidative stress

## Abstract

Plant responses to heat stress include complex transcriptional networks and protein regulations in which BTB/POZ-MATH (BPM) proteins participate as a part of ubiquitin-mediated protein degradation. *Arabidopsis thaliana* contains six *BPM* genes involved in responses to environmental changes, including heat. Seedlings overexpressing *BPM1* (*oeBPM1*), seedlings with downregulation of *BPM1*, *4*, *5*, and *6* (*amiR-bpm*) and wild type were exposed to 37 °C for 6 h. Treatment caused stronger decline of photosynthesis in *oeBPM1* than in *amiR-bpm* and wild type, although all seedlings recovered after 24 h at 24 °C. The activity of the antioxidant enzymes catalase, guaiacol peroxidase, and ascorbate peroxidase remained unchanged in *oeBPM1*, but increased in *amiR-bpm* and wild type. Heat stress induced HSP70 and HSP90 in all seedlings but expression remained notably higher in *amiR-bpm* after recovery. *DREB2A* and *HSFA3* expression increased in all seedlings immediately after stress, with the strongest induction in *amiR-bpm*. In *amiR-bpm* and wild type, *BPM2* expression was induced immediately after exposure, while *BPM1*, *BPM3*, *BPM4*, and *BPM6* were upregulated in wild type after recovery. In *oeBPM1* seedlings, *BPM4* expression decreased and *BPM6* expression increased immediately after treatment at 37 °C for 6 h. The results suggest that BPM proteins modulate heat stress response by influencing photosynthesis, activation of antioxidant enzymes, accumulation of HSPs, and expression of heat-responsive genes, thus contributing to the different physiological strategies observed in *A*. *thaliana* lines with altered expression of *BPM* genes.

## 1. Introduction

Proteostasis, or protein homeostasis, is essential for the maintenance of cellular function under optimal and stress conditions. The ubiquitin–proteasome system (UPS) is an important pathway that is responsible for selective protein degradation; it ensures the effective removal of misfolded, damaged, or unnecessary proteins [1]. Within this system, the BTB/POZ-MATH (BPM) proteins act as substrate adaptors that target specific proteins for degradation by the cullin3-RING E3 ubiquitin ligase complex [2]. Six *BPM* genes (*BPM1*-*6*) have been identified in *Arabidopsis thaliana* (L.) Heynh. and their functions are gradually being elucidated. BPM proteins regulate numerous developmental and stress-related processes by mediating the degradation of transcription factors, signalling proteins and enzymes. A study by Weber and Hellmann [3] showed that BPM proteins interact with transcription factors of the APETALA2/ethylene response factor (AP2/ERF) family, which play a crucial role in plant stress responses. The regulation of AP2/ERFs represents the final step in the ethylene signalling pathway, which controls the biosynthesis of important phytohormones such as ethylene, cytokinins, gibberellins, and abscisic acid (ABA) [4,5]. Further research revealed that BPM proteins interact directly with another transcription factor, the homeobox–leucine zipper protein ATHB6, a negative regulator of the ABA signalling pathway [6]. BPM proteins are also involved in fatty acid metabolism as they interact with the transcriptional activator wrinkled 1 (WRI1), an important regulator of lipid biosynthesis [7]. In addition, the BPM proteins influence the stability of MYB-domain protein 56 (MYB56), a negative regulator of flowering, further emphasizing their role in developmental control [8]. Recently, a novel function of the BPM1 protein beyond the UPS has been proposed, showing that it directly interacts with the proteins defective in meristem silencing 3 (DMS3) and RNA-directed DNA methylation 1 (RDM1), both of which are integral components of the RNA-directed DNA methylation (RdDM) mechanism [9]. *BPM1*-*6* transcripts have been detected in various organs of *A*. *thaliana* seedlings, including cotyledons, hypocotyls, and roots [10], and BPM proteins are expressed in different cell types such as guard cells, mesophyll cells, and epidermal root cells [6,11]. Their subcellular localisation in the nucleus and cytosol suggests their involvement in the regulation of various stress-related signalling pathways [3,12].

One of the major environmental factors affecting the survival and productivity of plants is heat stress. The global rise in temperature poses a serious threat to agricultural yields, as even moderately elevated temperatures can disrupt cellular homeostasis and cause irreversible damage to proteins and membranes [13,14,15]. Photosynthesis is one of the most heat-sensitive physiological processes, as it is highly dependent on the fluidity and functionality of membranes, protein complexes, and many enzymes. Photosystem II (PSII) is an important component of the photochemical reactions of photosynthesis and is very sensitive to elevated temperatures leading to a decrease in photosynthetic performance [16,17]. A widely used technique to assess PSII functionality is the induction of chlorophyll *a* (Chl *a*) fluorescence and analysis by the JIP-test. Measured and calculated parameters obtained by this method, such as the maximum quantum yield of PSII photochemistry (F_V_/F_M_) and the performance index on absorption basis (PI_ABS_), describe the overall efficiency of photosynthesis. Additionally, the specific energy fluxes associated with PSII activity include light absorption (ABS), trapping of excitation energy (TR_0_), conversion to electron transport (ET_0_), and dissipation of excess energy, mainly in the form of heat (DI_0_), all of which are generally expressed per active reaction centre (RC) of PSII [18,19]. Heat stress also affects the composition of photosynthetic pigments (chlorophylls and carotenoids), which can be a useful indication of the stress-induced changes [17,20]. Various abiotic and biotic stress factors, including elevated temperatures, lead to the formation of reactive oxygen species (ROS) [21]. The main source of ROS in plant cells are photosynthetic reactions in the chloroplasts, but ROS are also formed in other cell compartments and membranes. They are involved in many signalling pathways, but excessive production of ROS, especially hydrogen peroxide (H_2_O_2_) and superoxide radicals (O_2_^•−^), can cause oxidative damage to lipids, proteins, and nucleic acids, ultimately compromising cellular integrity [21,22,23]. To mitigate the accumulation of ROS, plants have developed complex antioxidant defence systems consisting of enzymatic and non-enzymatic components. Key antioxidant enzymes, including superoxide dismutase (SOD), catalase (CAT), ascorbate peroxidase (APX), and guaiacol peroxidase (G-POD), play a crucial role in neutralising ROS and preventing oxidative damage [21,24]. In addition, biochemical markers such as malondialdehyde (MDA), a by-product of lipid peroxidation, and proline, an osmoprotectant with ROS-scavenging properties, serve as indicators of oxidative stress level [24,25]. As signalling molecules, ROS can activate heat shock factors (HSFs), a family of transcription factors that regulate the response to heat stress [26,27]. Activated HSFs induce the expression of heat shock proteins (HSPs), molecular chaperones that aid in protein folding, prevent aggregation and facilitate the degradation of misfolded proteins. These protective mechanisms are essential for maintaining cellular homeostasis under heat stress, as uncontrolled protein misfolding and aggregation can lead to cellular dysfunction and ultimately cell death [28,29].

Despite the increasing knowledge about the function of BPM proteins, our understanding of their role in the adaptation of plants to heat stress is still limited. BPM proteins interact with dehydration-responsive element-binding protein 2A (DREB2A), an important transcription factor that regulates the response to heat and drought stress, suggesting that they may modulate stress adaptation by controlling DREB2A stability [12]. In particular, DREB2A plays a central role in the activation of heat-stress-responsive genes by inducing the transcription of *HSFA3*, which in turn regulates a broad network of stress-responsive genes [30,31]. Further evidence for the involvement of BPM proteins in the response to heat stress is that BPM1 is induced and remains stable at elevated temperatures, particularly at 37 °C [11]. Regardless of these findings, the broader physiological and biochemical functions of BPM proteins during heat stress and their regulatory mechanisms are still poorly understood. Considering their role in protein turnover, further research is needed to elucidate how BPM proteins contribute to heat stress adaptation, in particular their potential to modulate stress-responsive transcription factors, protein stability, oxidative stress balance, and photosynthetic performance.

This study aims to elucidate the role of BPM proteins in heat stress adaptation by analysing the physiological, biochemical, and molecular responses of *A. thaliana* seedlings with altered expression of *BPM* genes. The seedling stage is particularly sensitive to unfavourable temperatures and is therefore ideal for studying responses to heat stress, as it directly influences the subsequent survival and development of the plants [32]. Therefore, we investigated the following aspects: (1) photosynthetic performance, assessed by Chl *a* fluorescence parameters and pigment content; (2) oxidative stress markers, including H_2_O_2_, MDA, and proline, alongside the activity of key antioxidant enzymes (G-POD, APX, CAT, and SOD); and (3) the expression of heat-stress-responsive proteins (HSP70, HSP90) and genes (*DREB2A*, *HSFA3*, and *BPM1*-*6*) in *A. thaliana* seedlings with overexpression of the *BPM1* gene (*oeBPM1*) and seedlings with reduced expression of the *BPM1*, *4*, *5*, and *6* genes (*amiR-bpm*). We have shown that BPM proteins regulate the response of plants to heat stress by modulating photosynthetic performance, antioxidant defence, and stress-responsive proteins and genes. By elucidating their functional role under heat stress, this study contributes to the existing knowledge on the role of BPM proteins in proteostasis and thermotolerance, providing new insights into the adaptive strategies that plants use to cope with increasing temperatures.

## 2. Results

### 2.1. Photosynthetic Responses to Heat Stress in A. thaliana Lines with Altered BPMs Gene Expression

The impact of moderate heat stress (i.e., treated vs. control seedlings) and the tested lines (i.e., wild type vs. *oeBPM1* vs. *amiR-bpm* seedlings) on the photosynthetic efficiency of *A. thaliana* seedlings was assessed using the JIP-test. The focus was on the analysis of two fluorescence parameters describing photosynthetic efficiency—F_V_/F_M_ and PI_ABS_.

**Figure 1:**
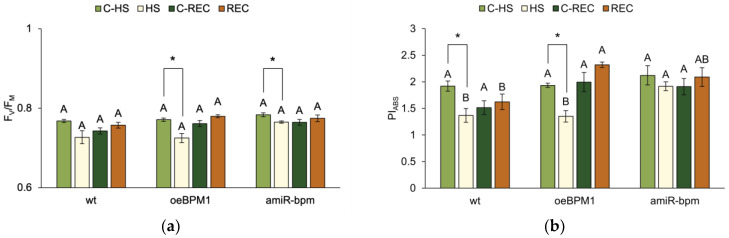
(**a**) Maximum quantum yield of PSII photochemistry (F_V_/F_M_) and (**b**) performance index on absorption basis (PI_ABS_) in *Arabidopsis thaliana* seedlings with *BPM1* overexpression (*oeBPM1*), seedlings with *BPM1*, *4*, *5*, and *6* downregulation (*amiR-bpm*), and wild-type (wt) seedlings. Measurements were taken at two time points: immediately after exposure to 37 °C for 6 h (HS) and after a 24 h recovery at 24 °C (REC). The control groups (C-HS and C-REC) were kept at 24 °C throughout the experiment. Data are expressed as mean ± standard error (n = 5 biological replicates). Differences between two groups—control and heat-treated seedlings sampled at the same time point (immediately after the treatment or after 24 h recovery, i.e., C-HS vs. HS, or C-REC vs. REC)—were determined using Student’s *t*-test, with significant differences (*p* ≤ 0.05) indicated by asterisk (*). For comparisons of the tested lines (wild type vs. *oeBPM1* vs. *amiR-bpm*) exposed to the same treatment condition (C-HS, HS, C-REC, or REC), a one-way ANOVA followed by Tukey’s HSD post hoc test was performed, with significant differences (*p* ≤ 0.05) indicated by different uppercase letters.

A two-way ANOVA revealed that only heat treatment significantly affected F_V_/F_M_ immediately after heat exposure (F = 23.6, *p* ≤ 0.0001). Accordingly, treated *oeBPM1* and *amiR-bpm* seedlings had significantly lower F_V_/F_M_ than the control seedlings of these lines (Figure 1a). In contrast, wild-type seedlings showed no significant change in F_V_/F_M_ after heat stress. PI_ABS_ measured immediately after heat treatment differed significantly between control and treated seedlings (F = 22.83, *p* ≤ 0.0001) and between the tested lines (F = 7.14, *p* ≤ 0.01). In *oeBPM1* and wild-type seedlings, PI_ABS_ was significantly reduced in response to heat stress, while no significant changes were observed in *amiR-bpm* seedlings compared to the corresponding control (Figure 1b). When comparing heat-treated seedlings of different lines, *amiR-bpm* seedlings showed significantly higher PI_ABS_ levels than *oeBPM1* and wild-type seedlings. Under control conditions, no significant differences were found between the three lines.

After a 24 h recovery period, F_V_/F_M_ did not differ significantly between control and treated seedlings or between the tested lines. In contrast, the tested lines remained a significant factor influencing PI_ABS_ (F = 8.76, *p* ≤ 0.01). Accordingly, no significant differences were found between control and treated seedlings in all three lines (Figure 1b). However, when comparing the heat-treated seedlings of three tested lines, the *oeBPM1* seedlings showed a significantly higher PI_ABS_ value than the wild type (Figure 1b), while the *amiR-bpm* seedlings showed no significant differences compared to the other two lines.

### 2.2. Specific Energy Fluxes in Photosystem II

To further investigate the observed changes in photosynthetic efficiency, four key parameters of energy flux contributing to PI_ABS_ were analysed—ABS/RC, TR_0_/RC, ET_0_/RC, and DI_0_/RC.

Significant changes in ABS/RC observed immediately after heat stress were caused by heat treatment (F = 5.98, *p* ≤ 0.05), tested lines (F = 4.93, *p* ≤ 0.05), and their interactions (F = 4.26, *p* ≤ 0.05). Consequently, a significant increase in ABS/RC was observed in heat-treated *oeBPM1* seedlings, while no significant changes were observed in *amiR-bpm* or wild-type seedlings after heat exposure, compared to the corresponding control (Table 1). When comparing the control seedlings of all three lines, *oeBPM1* and *amiR-bpm* showed significantly lower ABS/RC than the wild type. However, no significant differences were found between the heat-treated seedlings of the tested lines. For TR_0_/RC and ET_0_/RC, a significant interaction was observed between heat treatment and the tested lines (TR_0_/RC: F = 6.74, *p* ≤ 0.01; ET_0_/RC: F = 12.21, *p* ≤ 0.001). A significant reduction in both parameters was observed in heat-treated wild-type seedlings, while no significant differences were observed when comparing the heat-treated and control seedlings of the *oeBPM1* and *amiR-bpm* lines (Table 1). When comparing the control seedlings of three tested lines, both *oeBPM1* and *amiR-bpm* seedlings showed significantly lower TR_0_/RC and ET_0_/RC values than the wild type. A significant effect of heat treatment (F = 15.87, *p* ≤ 0.001) and tested lines (F = 5.42, *p* ≤ 0.05) was also found for DI_0_/RC. Heat-treated *oeBPM1* and *amiR-bpm* seedlings had significantly higher DI_0_/RC compared to the corresponding controls (Table 1), while no significant changes were observed in wild-type seedlings. The comparison of the control seedlings of three tested lines revealed that the *oeBPM1* and *amiR-bpm* seedlings had a significantly lower DI_0_/RC value than the wild-type seedlings.

After a 24 h recovery period, ABS/RC was significantly affected by the tested lines (F = 7.14, *p* ≤ 0.01). Thus, when comparing heat-treated and control seedlings, no significant changes were observed, regardless of the line tested (Table 1). However, when comparing the heat-treated seedlings of three *A*. *thaliana* lines, *oeBPM1* showed significantly lower ABS/RC than the wild type. For TR_0_/RC and ET_0_/RC, two-way ANOVA showed no significant effect of heat treatment, the lines tested or their interaction after recovery. In contrast, the tested lines had a significant effect on DI_0_/RC (F = 8.80, *p* ≤ 0.01). Accordingly, no significant differences in DI_0_/RC were found when comparing heat-treated and control seedlings (Table 1). However, when comparing the lines, the DI_0_/RC of the heat-treated *oeBPM1* and *amiR-bpm* seedlings were significantly lower than those of the wild type.

### 2.3. Impact of Heat Stress and Modified BPMs Gene Expression on Pigment Composition

In addition to chlorophyll fluorescence, we measured pigment content in wild-type, *oeBPM1*, and *amiR-bpm* seedlings to gain a deeper understanding of how the different tested lines affected the photosynthetic performance of the plants under both stress and optimal conditions.

The Chl *a* content measured immediately after heat treatment was significantly influenced by the interaction between heat stress and the tested lines (F = 4.41, *p* ≤ 0.05). A significant reduction in Chl *a* content was observed in heat-treated *oeBPM1* seedlings, while no significant changes were observed in heat-treated *amiR-bpm* or wild-type seedlings compared to the respective controls (Table 2). Comparisons between the control seedlings of all three lines showed that *oeBPM1* seedlings had a significantly higher Chl *a* content than *amiR-bpm* seedlings. Wild-type seedlings did not differ significantly from the two transgenic lines. Chlorophyll *b* (Chl *b*) content remained unchanged in all three *A. thaliana* lines, with no significant effects of heat treatment, the lines tested, or their interactions (Table 2). In contrast, the interaction between heat stress and the tested lines (F = 4.38, *p* ≤ 0.05) had a significant effect on the total carotenoids (Cars) content immediately after heat exposure. Accordingly, a significant decrease in Cars content was observed in heat-treated *oeBPM1* and wild-type seedlings compared to their respective controls (Table 2). When comparing the control seedlings of the three lines tested, the *oeBPM1* seedlings showed a significantly higher Cars content than the *amiR-bpm* seedlings, while the wild-type seedlings did not differ significantly from the other two lines.

After a recovery period of 24 h, the heat treatment, the tested lines and their interactions had no significant effect on any of the pigments analysed.

### 2.4. Contribution of Heat Stress and Modified BPMs Gene Expression to Oxidative Stress Levels

To assess heat-induced cell damage in wild-type, *oeBPM1*, and *amiR-bpm* seedlings, the level of oxidative stress was determined by measuring characteristic biochemical markers—H_2_O_2_, MDA, and proline content.

**Figure 2:**
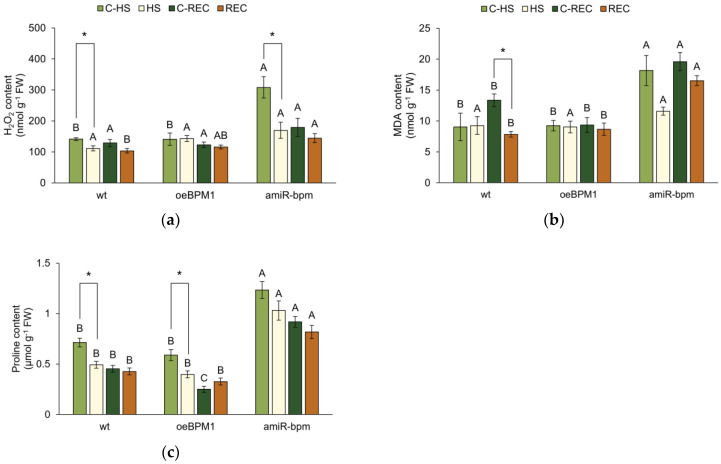
(**a**) Hydrogen peroxide (H_2_O_2_), (**b**) malondialdehyde (MDA), and (**c**) proline content in *Arabidopsis thaliana* seedlings with *BPM1* overexpression (*oeBPM1*), seedlings with *BPM1*, *4*, *5*, and *6* downregulation (*amiR-bpm*), and wild-type (wt) seedlings. Samples were collected at two time points: immediately after exposure to 37 °C for 6 h (HS) and after a 24 h recovery at 24 °C (REC). The control groups (C-HS and C-REC) were kept at 24 °C throughout the experiment. Data are expressed as mean ± standard error (n = 5 biological replicates). Differences between two groups—control and heat-treated seedlings sampled at the same time point (immediately after the treatment or after 24 h recovery, i.e., C-HS vs. HS, or C-REC vs. REC)—were determined using Student’s *t*-test, with significant differences (*p* ≤ 0.05) indicated by asterisk (*). For comparisons of the tested lines (wild type vs. *oeBPM1* vs. *amiR-bpm*) exposed to the same treatment condition (C-HS, HS, C-REC, or REC), a one-way ANOVA followed by Tukey’s HSD post hoc test was performed, with significant differences (*p* ≤ 0.05) indicated by different uppercase letters.

Immediately after heat stress, H_2_O_2_ content was significantly affected by heat treatment (F = 10.64, *p* ≤ 0.01), the tested lines (F = 17.20, *p* ≤ 0.0001), and their interactions (F = 6.37, *p* ≤ 0.01). Heat-treated *amiR-bpm* and wild-type seedlings showed a significant reduction in H_2_O_2_ content, while no significant changes were observed in treated *oeBPM1* seedlings compared to the respective controls (Figure 2a). In addition, the difference between the control seedlings and the heat-treated seedlings was more pronounced in the *amiR-bpm* line than in the wild type. The comparison of the control seedlings of the three tested lines showed that the *amiR-bpm* seedlings had a significantly higher H_2_O_2_ content than the *oeBPM1* and wild-type seedlings. On the other hand, the heat-treated seedlings of all three tested lines showed no significant differences. The level of lipid peroxidation, expressed as MDA content, was significantly affected only by the tested lines (F = 8.79, *p* ≤ 0.01). Consequently, no significant differences in MDA content were observed between the control and the heat-treated seedlings, regardless of the line tested (Figure 2b). Although the difference was not statistically significant, heat-treated *amiR-bpm* seedlings had a 1.6-fold lower MDA content compared to the corresponding control. When comparing control seedlings of different lines, *amiR-bpm* seedlings had significantly higher MDA levels than *oeBPM1* and wild-type seedlings. Immediately after heat treatment, the proline content was significantly affected by both the heat treatment (F = 16.49, *p* ≤ 0.001) and the tested lines (F = 60.90, *p* ≤ 0.0001). A significant reduction in proline content was observed in heat-treated *oeBPM1* and wild-type seedlings, while no significant changes were observed in the heat-treated *amiR-bpm* seedlings compared to the respective control (Figure 2c). When comparing the lines, both the heat-treated and control *amiR-bpm* seedlings had significantly higher proline content than the *oeBPM1* and wild-type seedlings.

After a 24 h recovery period, only the tested lines had a significant effect on the H_2_O_2_ content (F = 5.74, *p* ≤ 0.01). Accordingly, no significant differences in H_2_O_2_ content were observed between control and heat-treated seedlings, regardless of the line tested (Figure 2a). Although not statistically significant, the H_2_O_2_ content in heat-treated wild-type seedlings was 1.3-fold lower compared to the corresponding control. When comparing the control seedlings of the different lines, no significant differences in H_2_O_2_ content were found. However, when comparing the heat-treated seedlings, the *amiR-bpm* seedlings had a significantly higher H_2_O_2_ content than the wild type. MDA content was significantly affected by both the heat treatment (F = 12.36, *p* ≤ 0.01) and the tested lines (F = 41.49, *p* ≤ 0.0001). A significant reduction in MDA content was observed in heat-treated wild-type seedlings compared to the corresponding control (Figure 2b). Interestingly, a comparison of the lines showed that both the control and heat-treated *amiR-bpm* seedlings had significantly higher MDA content than the *oeBPM1* and wild-type seedlings. After the recovery period, the tested lines continued to have a significant influence (F = 94.03, *p* ≤ 0.0001) on the proline content. Accordingly, no significant differences were found between the heat-treated and control seedlings, regardless of the line tested (Figure 2c). Although not statistically significant, the proline content in heat-treated *oeBPM1* seedlings increased 1.3-fold compared to the corresponding control. When comparing the lines, the analysis showed that both the heat-treated and control seedlings of *amiR-bpm* had significantly higher proline levels than the *oeBPM1* and wild-type seedlings. In addition, the *oeBPM1* control seedlings had a significantly lower proline content than the wild type.

### 2.5. Antioxidant Activity in Response to Heat Stress and Modified BPMs Gene Expression

To assess the plant’s ability to attenuate oxidative stress and prevent cell damage, the activity of four antioxidant enzymes—G-POD, APX, CAT, and SOD—was measured.

**Figure 3:**
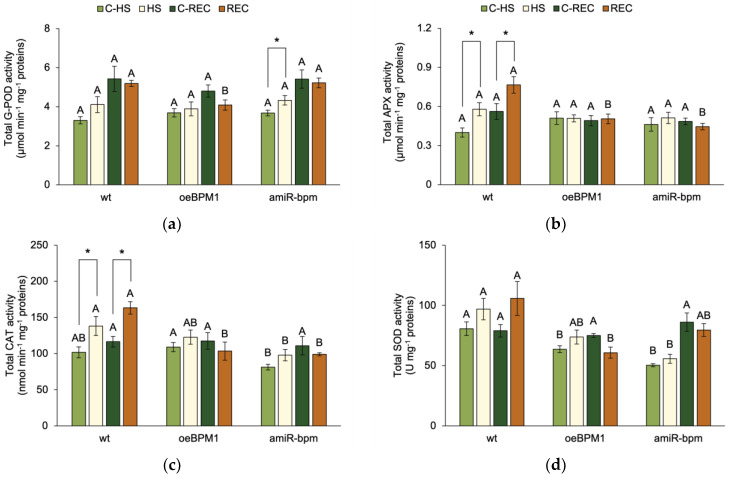
The total activity of (**a**) guaiacol peroxidase (G-POD), (**b**) ascorbate peroxidase (APX), (**c**) catalase (CAT) and (**d**) superoxide dismutase (SOD) in *Arabidopsis thaliana* seedlings with *BPM1* overexpression (*oeBPM1*), seedlings with *BPM1*, *4*, *5*, and *6* downregulation (*amiR-bpm*), and wild-type (wt) seedlings. Samples were collected at two time points: immediately after exposure to 37 °C for 6 h (HS) and after a 24 h recovery at 24 °C (REC). The control groups (C-HS and C-REC) were kept at 24 °C throughout the experiment. Data are expressed as mean ± standard error (n = 5 biological replicates). Differences between two groups—control and heat-treated seedlings sampled at the same time point (immediately after the treatment or after 24 h recovery, i.e., C-HS vs. HS, or C-REC vs. REC)—were determined using Student’s *t*-test, with significant differences (*p* ≤ 0.05) indicated by asterisk (*). For comparisons of the tested lines (wild type vs. *oeBPM1* vs. *amiR-bpm*) exposed to the same treatment condition (C-HS, HS, C-REC, or REC), a one-way ANOVA followed by Tukey’s HSD post hoc test was performed, with significant differences (*p* ≤ 0.05) indicated by different uppercase letters.

Immediately after heat exposure, G-POD activity was significantly influenced by the treatment (F = 5.83, *p* ≤ 0.05). A significant increase in G-POD activity was observed in heat-treated *amiR-bpm* seedlings compared to the corresponding control (Figure 3a). In contrast, no significant changes were observed in treated *oeBPM1* and wild-type seedlings. Although not statistically significant, G-POD activity increased 1.3-fold in treated wild-type seedlings compared to the control. APX activity was also significantly affected by heat treatment (F = 4.54, *p* ≤ 0.05), while CAT activity was influenced by both heat treatment (F = 10.0, *p* ≤ 0.01) and the tested lines (F = 7.32, *p* ≤ 0.01). A significant increase in APX and CAT activity was observed in the treated wild-type seedlings compared to the corresponding control (Figure 3b,c). In contrast, no significant differences were observed between the heat-treated and control seedlings of the *oeBPM1* and *amiR-bpm* lines, although CAT activity increased 1.3-fold in the treated *amiR-bpm* seedlings. When comparing enzyme activity between lines, CAT activity was significantly lower in the *amiR-bpm* control seedlings than in the *oeBPM1* control seedlings. In addition, heat-treated *amiR-bpm* seedlings had significantly lower CAT activity than treated wild-type seedlings. SOD activity was significantly influenced by the tested lines (F = 22.81, *p* ≤ 0.0001). Accordingly, no significant differences were found between the heat-treated and control seedlings, regardless of the line tested (Figure 3d). Although not statistically significant, SOD activity increased 1.3-fold in heat-treated wild-type seedlings compared to the control. When comparing the control seedlings of the three lines, *oeBPM1* and *amiR-bpm* seedlings showed significantly lower SOD activity than the wild-type seedlings. In the heat-treated seedlings, SOD activity was significantly lower in *amiR-bpm* seedlings compared to the wild type.

After the 24 h recovery period, only the tested lines had a significant effect on G-POD (F = 3.53, *p* ≤ 0.05) and SOD activity (F = 5.41, *p* ≤ 0.05). Consequently, no significant differences in enzyme activities were observed between the control seedlings and the heat-treated seedlings, regardless of the line tested (Figure 3a,d). However, heat-treated wild-type seedlings showed a 1.4-fold higher SOD activity compared to the control. When comparing enzyme activity between lines, G-POD and SOD activity was significantly lower in heat-treated *oeBPM1* seedlings compared to wild-type seedlings, and G-POD activity was also lower in *oeBPM1* seedlings than in *amiR-bpm* seedlings. The activity of APX and CAT was significantly influenced by the tested lines (APX: F = 10.78, *p* ≤ 0.001; CAT: F = 5.52, *p* ≤ 0.05) and the interaction between treatment and tested lines (APX: F = 3.91, *p* ≤ 0.05; CAT: F = 4.72, *p* ≤ 0.05). Both APX and CAT activity were significantly higher in the heat-treated wild-type seedlings compared to the respective control (Figure 3b,c). However, no significant differences were observed between the heat-treated and control seedlings of the *oeBPM1* and *amiR-bpm* lines. When comparing the seedlings between lines, the treated *oeBPM1* and *amiR-bpm* seedlings showed significantly lower APX and CAT activity than the treated wild-type seedlings.

### 2.6. Heat-Responsive Proteins and Genes in Seedlings with Altered BPMs Expression

To investigate the activation of heat stress response mechanisms in wild-type, *oeBPM1*, and *amiR-bpm* seedlings, we measured the expression of HSP70 and HSP90 proteins as well as *DREB2A*, *HSFA3*, and *BPM* genes.

**Figure 4:**
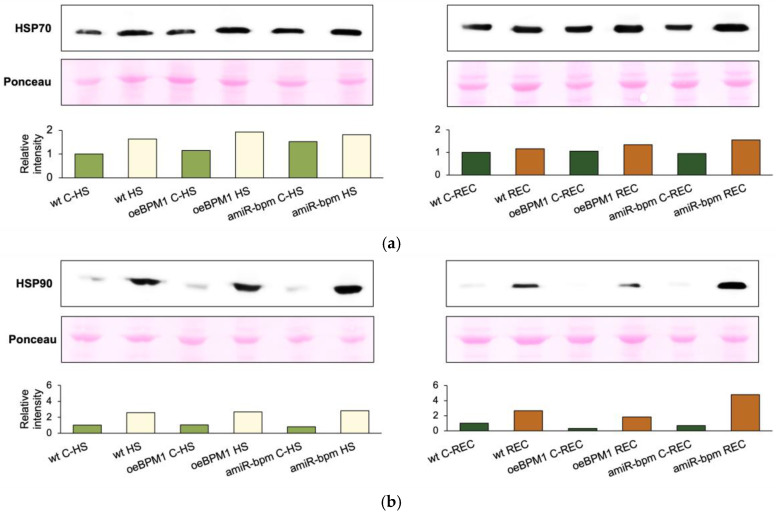
Immunodetection of (**a**) HSP70 and (**b**) HSP90 proteins in *Arabidopsis thaliana* seedlings with *BPM1* overexpression (*oeBPM1*), seedlings with *BPM1*, *4*, *5*, and *6* downregulation (*amiR-bpm*), and wild-type (wt) seedlings. Samples were collected at two time points: immediately after exposure to 37 °C for 6 h (HS) and after a 24 h recovery at 24 °C (REC). The control groups (C-HS and C-REC) were kept at 24 °C throughout the experiment. Ponceau staining of the respective lane for each sample was used as a qualitative loading and transfer control (only a portion of the stained membrane was shown). The graphical representation shows the relative band intensity of HSP70 and HSP90 normalised to the wt C-HS and wt C-REC (set as 1). Immunodetection was performed on three biological replicates, with one representative biological replicate selected for presentation.

Immediately after heat stress, HSP70 protein expression increased 1.7-fold in heat-treated *oeBPM1* seedlings and 1.6-fold in wild-type seedlings, compared to the respective controls (Figure 4a). Conversely, heat-treated *amiR-bpm* seedlings showed no notable increase in HSP70 expression immediately after heat treatment. Interestingly, the comparison between the control seedlings of the three lines revealed that *amiR-bpm* seedlings had a 1.5-fold higher HSP70 expression than the wild-type control seedlings at the first time point. In contrast to HSP70, the expression of HSP90 was strongly induced in all heat-treated seedlings. Expression increased 2.6-fold in both *oeBPM1* and wild-type seedlings, while a 3.5-fold increase was observed in *amiR-bpm* seedlings (Figure 4b).

After a 24 h recovery period at 24 °C, HSP70 expression was increased 1.6-fold in heat-treated *amiR-bpm* seedlings compared to the corresponding control (Figure 4a). In contrast, HSP70 levels returned to control levels in both *oeBPM1* and wild-type seedlings during the recovery period. HSP90 expression continued to increase during the recovery period, reaching a 5.8-fold higher level in *oeBPM1* and 6.8-fold higher level in *amiR-bpm* seedlings, while it remained at a 2.7-fold higher level in wild-type seedlings compared to the corresponding controls (Figure 4b). Comparison of the lines after recovery showed that the *oeBPM1* control seedlings had a 3.2-fold lower HSP90 expression than the wild-type control seedlings.

**Figure 5:**
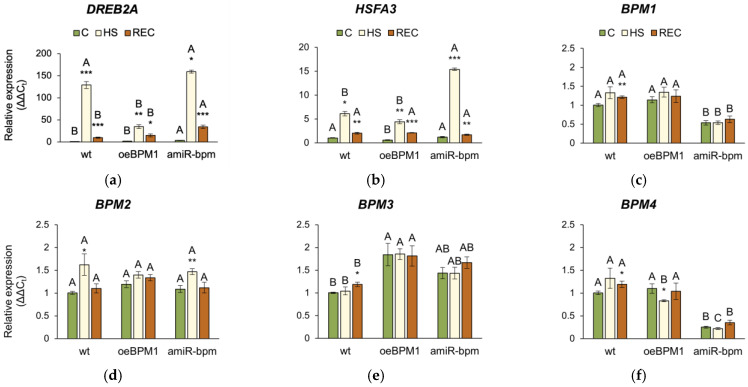

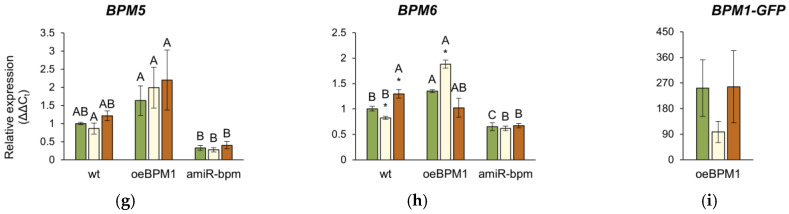
Relative expression of (**a**) *DREB2A*, (**b**) *HSFA3*, (**c**) *BPM1*, (**d**) *BPM2*, (**e**) *BPM3*, (**f**) *BPM4*, (**g**) *BPM5*, (**h**) *BPM6*, and (**i**) *BPM1-GFP* in *Arabidopsis thaliana* seedlings. All gene expressions, except the *BPM1-GFP* transgene, were analysed in seedlings with *BPM1* overexpression (*oeBPM1*), seedlings with *BPM1*, *4*, *5*, and *6* downregulation (*amiR-bpm*) and wild-type (wt) seedlings. Transgene expression (*BPM1-GFP*) was only measured in the *oeBPM1* line. Seedlings were collected before exposure to 37 °C (C), immediately after exposure to 37 °C (HS), and after 24 h recovery at 24 °C (REC). Results are presented as relative mean of three biological replicates ± standard error, normalized to wt C (set as 1). Asterisks show significant differences (Student’s *t*-test) between C and HS or C and REC for each line at *p* ≤ 0.05 (*), *p* ≤ 0.01 (**) and *p* ≤ 0.001 (***). For comparisons of tested lines (wild type vs. *oeBPM1* vs. *amiR-bpm*) exposed to the C, HS, or REC, a one-way ANOVA followed by Tukey’s HSD post hoc test was performed, with significant differences (*p* ≤ 0.05) indicated by different uppercase letters.

The expression of *DREB2A* and *HSFA3* was significantly influenced by heat exposure (*DREB2A*: F = 17.38, *p* ≤ 0.001; *HSFA3*: F = 39.22, *p* ≤ 0.001), tested lines (*DREB2A*: F = 8.72, *p* ≤ 0.001; *HSFA3*: F = 20.26, *p* ≤ 0.001), and their interactions (*DREB2A*: F = 13.47, *p* ≤ 0.001; *HSFA3*: F = 45.13, *p* ≤ 0.001). Accordingly, the expression of both *DREB2A* and *HSFA3* was significantly upregulated in all heat-treated seedlings immediately after heat stress compared to the respective controls (Figure 5a,b). Comparison of the three lines showed that the induction of *DREB2A* was more pronounced in *amiR-bpm* and wild-type seedlings immediately after heat exposure than in *oeBPM1*. Although *DREB2A* expression decreased after recovery in all three lines, it remained higher than in control seedlings, with *amiR-bpm* seedlings showing the highest expression. The expression of *HSFA3* followed a similar pattern and showed significant upregulation in response to heat stress in all three lines compared to the corresponding control. The highest induction immediately after exposure was observed in *amiR-bpm* seedlings. Although *HSFA3* expression remained significantly increased after recovery, no differences were observed between the lines tested.

The relative expression of the endogenous *BPM1*-*6* genes was analysed in all three lines. Additionally, in the *oeBPM1* line, which contains *BPM1-GFP* transgene, the expression of the transgene was also evaluated. Considering both time points, two-way ANOVA revealed that heat treatment significantly affected the expression of five *BPM* genes—*BPM1* (F = 28.92, *p* ≤ 0.001), *BPM2* (F = 15.84, *p* ≤ 0.001), *BPM3* (F = 9.56, *p* ≤ 0.01), *BPM4* (F = 2.73, *p* ≤ 0.05), and *BPM6* (F = 23.44, *p* ≤ 0.001). Significant changes in the expression of *BPM2*, *BPM4*, and *BPM6* were observed immediately after heat stress. Specifically, the expression of *BPM2* increased significantly in heat-treated *amiR-bpm* and wild-type seedlings, while no significant changes were observed in *oeBPM1* seedlings compared to the respective control (Figure 5d). Conversely, *BPM4* expression decreased significantly in heat-treated *oeBPM1* seedlings, while *BPM6* expression increased in the same seedlings compared to the control seedlings (Figure 5f,h). In contrast, *BPM6* expression decreased significantly in heat-treated wild-type seedlings compared to the corresponding control. After the 24 h recovery period, significant changes in *BPM*s gene expression were observed only in heat-treated wild-type seedlings, where a significant upregulation of *BPM1*, *BPM3*, *BPM4*, and *BPM6* was observed compared to control values (Figure 5c,e,f,h). The expression of *BPM5* remained unchanged in all analysed lines compared to the respective controls (Figure 5g). Although the expression of the *BPM1*-*GFP* transgene decreased 2.58-fold immediately after heat stress, the change was not statistically significant compared to the control values (Figure 5i). When comparing *BPM*s expression between control seedlings of the three lines confirmed that *amiR-bpm* seedlings had reduced expression of *BPM1*, *BPM4*, *BPM5*, and *BPM6* compared to *oeBPM1* and wild-type seedlings. Interestingly, the wild-type control seedlings showed lower expression of *BPM3* and *BPM6* than the *oeBPM1* seedlings.

## 3. Discussion

*A*. *thaliana* seedlings overexpressing the *BPM1* gene (*oeBPM1*) and seedlings with reduced expression of the *BPM1*, *4*, *5*, and *6* genes (*amiR-bpm*) were used as plant models to investigate the role of BPM proteins in plant response to moderate heat stress (37 °C for 6 h). Different stress strategies were revealed that can be attributed to the balance of stress perception, signal transduction, and proteostasis regulation.

### 3.1. BPM1 Overexpression Increases the Sensitivity of Photosynthesis to Heat Stress

In recent years, numerous studies have confirmed that photosynthesis is one of the physiological processes most sensitive to heat. The decline in photosynthetic efficiency often occurs at temperatures lower than those affecting other physiological processes [17,33,34]. In our study, the measurement of Chl *a* fluorescence using the JIP-test generally showed the influence of heat on the photosynthetic performance and functionality of PSII. The parameter F_V_/F_M_, which indicates the maximum quantum efficiency of PSII photochemistry, is widely recognized as a reliable marker of heat-induced inhibition of PSII activity [35,36,37,38]. Under non-stress conditions, F_V_/F_M_ values are typically between 0.7 and 0.83 [39]. Consistently, all control seedlings, including the transgenic lines and the wild type, showed values in this range, indicating optimal activity of PSII. However, the heat-treated *oeBPM1* and *amiR-bpm* seedlings showed a significant decrease in F_V_/F_M_ immediately after heat exposure, while the wild-type seedlings showed a similar trend, although the difference was not significant. Another parameter derived from the JIP-test, PI_ABS_, serves as a comprehensive indicator of the vitality of the photosynthetic apparatus and quantifies the efficiency with which the absorbed light energy is utilised in photochemical reactions [35,40]. In the present study, PI_ABS_ was significantly reduced in both *oeBPM1* and wild-type seedlings after heat stress. Furthermore, the simultaneous and significant decrease in PI_ABS_ and F_V_/F_M_ in the *oeBPM1* line indicates an increased sensitivity of the photosynthetic machinery to moderate heat stress. In contrast, only F_V_/F_M_ was reduced in the *amiR-bpm* line and only PI_ABS_ in the wild type suggesting that the extent of photosynthetic impairment was more pronounced in the *oeBPM1* seedlings. This observation is consistent with the results in wild barley (*Hordeum spontaneum*), where heat-sensitive cultivars showed significantly lower F_V_/F_M_ and PI_ABS_ values under stress than more heat-tolerant genotypes [41].

To further investigate the effects of heat stress on photosynthetic efficiency, four parameters of energy fluxes contributing to PI_ABS_ were evaluated: absorption (ABS/RC), energy trapping (TR_0_/RC), electron transport (ET_0_/RC), and energy dissipation (DI_0_/RC). Increased ABS/RC and DI_0_/RC values were observed in *oeBPM1* seedlings immediately after heat treatment, indicating higher energy absorption and dissipation. Such trends have been documented in plants exposed to elevated temperatures [42,43]. According to Strasser et al. [35], the increase in ABS/RC and DI_0_/RC in heat-treated plants is generally attributed to the partial inactivation of RCs, which leads to a greater energy load per remaining active RCs and increased energy dissipation as a protective mechanism. In addition, the increased ABS/RC and DI_0_/RC levels observed in heat-treated *oeBPM1* seedlings could also be a consequence of reduced pigment content. A decrease in photosynthetic pigments limits the light-harvesting capacity of the plant, which reduces photosynthetic efficiency and increases the dissipation of energy absorbed per functional RC [44,45]. Indeed, a significant reduction in pigment content was observed in *oeBPM1* seedlings immediately after heat exposure, supporting the observed increases in ABS/RC and DI_0_/RC. Interestingly, the *amiR-bpm* line, characterised by downregulation of *BPM1*, *4*, *5*, and *6* [6], showed a more stable photosynthetic response, suggesting that depletion of *BPM*s expression may have protective effects. These results are consistent with those of Morimoto et al. [12], who reported improved survival and chlorophyll retention in *A. thaliana* plants with silenced *BPM1*-*6* genes under severe heat stress. It has been suggested that BPM proteins, particularly BPM2, influence the response to heat stress by negatively regulating the transcription factor DREB2A—a key regulator of thermotolerance [12]. In our study, 98-fold overexpression of *BPM1* in the *oeBPM1* line was associated with significantly decreased expression of *DREB2A* and its downstream target *HSFA3* immediately after heat exposure, compared to *amiR-bpm* and wild-type seedlings. Since HSFA3 regulates the expression of several heat shock proteins and stress-related genes [29,30,31], its decreased expression could impair the activation of protective mechanisms during heat stress. In other words, the reduced expression of *DREB2A* and *HSFA3* in *oeBPM1* seedlings may have impaired the induction of chaperones and antioxidant defence mechanisms, ultimately rendering the photosynthetic apparatus more susceptible to damage and contributing to the observed decrease in F_V_/F_M_, PI_ABS_, and pigment content. However, after a 24 h recovery period, photosynthetic parameters returned to control values in all lines, supporting the idea that exposure to 37 °C represents a moderate and reversible stress for *A. thaliana*.

### 3.2. Overexpression of BPM1 Reduces Activation of Antioxidant Defence

Heat stress often leads to secondary stress in plant cells, known as oxidative stress, which results from the overproduction of ROS and the subsequent disruption of cellular redox homeostasis [46,47]. Therefore, to determine whether moderately elevated temperature induces oxidative stress and to evaluate the antioxidant capacity in *A. thaliana* seedlings with altered *BPM*s expression, the levels of H_2_O_2_, MDA, and proline as well as the activity of key antioxidant enzymes including G-POD, APX, CAT, and SOD were measured.

Among the ROS produced, H_2_O_2_ is one of the most studied molecules due to its relative stability and its dual role in plant cells, both as a signalling molecule and as a potential source of oxidative damage [21,29,48]. In this study, a significant reduction in H_2_O_2_ content was observed in heat-treated *amiR-bpm* and wild-type seedlings, while no significant change was observed in *oeBPM1* seedlings, suggesting a BPM1-related response to oxidative stress. Previous studies have shown that H_2_O_2_ accumulation is typically more pronounced under severe heat stress (above 40 °C), while moderate stress (around 35–37 °C) does not significantly increase ROS levels [49]. Moreover, activation of the antioxidant defence system is considered to be a crucial mechanism by which plants mitigate ROS-induced cell damage [50,51]. Increased activity of enzymes involved in H_2_O_2_ scavenging—namely G-POD, APX, and CAT—was detected in both *amiR-bpm* and wild-type seedlings immediately after heat treatment. These results indicate that the applied moderate heat stress (37 °C for 6 h) was sufficient to induce antioxidant defence in these lines and thus prevent excessive ROS accumulation. This interpretation is further supported by the MDA content, a commonly used indicator of lipid peroxidation and membrane damage during oxidative stress [52]. No significant increase in MDA content was observed in any of the *BPM*-modified lines or in the wild type, suggesting that cell membranes remained largely protected during heat stress. Of particular note was the response of the *oeBPM1* line, in which neither the H_2_O_2_ and MDA content nor the activity of antioxidant enzymes changed significantly after heat treatment, indicating that no oxidative stress was induced under the conditions tested. It is possible that the decreased expression of *DREB2A* and *HSFA3* in the *oeBPM1* seedlings impaired the activation of these downstream antioxidant defence mechanisms, but the basal antioxidant capacity appeared to be sufficient to prevent oxidative damage under the mild stress conditions used. Alternatively, the absence of oxidative stress symptoms in *oeBPM1* could represent an adaptive response in which photosynthetic activity is downregulated to limit ROS production. As previously discussed, photosynthesis in this line was significantly reduced after heat stress, possibly minimising the generation of ROS and thus reducing the need for an enhanced antioxidant response. After recovery, photosynthetic activity returned to normal levels, further supporting the notion of a transient and regulated response to moderate heat stress. As for SOD activity, no significant changes in activity were observed in any of the lines, either immediately after heat exposure or after recovery. As SOD catalyses the conversion of O_2_^•−^ to H_2_O_2_, its stable activity is consistent with the observed H_2_O_2_ dynamics, which were either unchanged or reduced in all lines. Similar results were reported for garlic (*Allium sativum*) exposed to 35 °C, where no significant increase in SOD activity was observed until the plants were exposed to a higher temperature [53]. These results also confirm that the 37 °C applied in this study represents moderate heat stress.

In addition to the enzymatic defence mechanisms, proline accumulation was monitored as a non-enzymatic marker of oxidative stress. Proline has multiple functions under different stress conditions, including osmoregulation, free radical scavenging, protein stabilisation, and maintenance of cellular redox balance [54,55,56]. Contrary to expectations, a significant decrease in proline content was observed in heat-treated *oeBPM1* and wild-type seedlings, indicating that the applied heat treatment did not promote proline synthesis. Similar results were observed in cotton (*Gossypium hirsutum*) and wheat (*Triticum aestivum*) under comparable thermal conditions [49,57]. The reduction in proline levels after heat stress could be due to an energy shift in the plant cells. As the efficiency of photosynthesis decreases under heat stress, the energy balance within the cell changes. Proline metabolism is closely linked to cellular energy regulation, in particular the NAD^+^/NADH and NADP^+^/NADPH couples, which are crucial for energy transfer during stress responses [58]. Essentially, the NADH and NADPH produced during proline degradation could be used for various metabolic processes, including energy production and redox balance maintenance, especially under heat stress conditions.

Interestingly, the *amiR-bpm* line showed increased levels of H_2_O_2_, MDA, and proline under control conditions. These differences in baseline levels of these parameters compared to *oeBPM1* and wild type suggest that downregulation of *BPM1*, *4*, *5*, and *6* may disrupt proteostasis and redox homeostasis, leading to mild physiological stress even under optimal growth conditions. The increased MDA content was likely a consequence of the increased H_2_O_2_ level, as lipid peroxidation is a known result of ROS accumulation [51,52]. One possible explanation for the increased H_2_O_2_ content is an altered regulation of glycolate oxidase (GOX), the key enzyme responsible for H_2_O_2_ production in photosynthetic tissues [59]. Bauer et al. [60] identified GOX2 as a potential interaction partner of BPM1 by tandem affinity purification and mass spectrometry experiments. Of the three known GOX isoforms (GOX1-3) in *A*. *thaliana*, GOX2 is the most efficient H_2_O_2_ producer [61]. Since BPM proteins act as substrate adaptors in the ubiquitin–proteasome system and promote the degradation of specific proteins, it is plausible that reduced *BPM* expression in the *amiR-bpm* line impairs GOX2 turnover and thereby increases H_2_O_2_ production. However, this hypothesis needs to be further validated. In parallel, the increased proline content in *amiR-bpm* supports the findings of Vitko et al. [62], who proposed that the BPM1–DREB2A interaction regulates proline metabolism. DREB2A is known to suppress proline catabolism by downregulating enzymes such as proline dehydrogenase and prolyl 4-hydroxylase, as shown in transgenic *Robinia pseudoacacia* plants overexpressing *DREB2A* [63]. Therefore, the greatly increased *DREB2A* expression in *amiR-bpm* likely inhibited proline degradation, leading to its accumulation. Despite the increased levels H_2_O_2_ and MDA, no increase in antioxidant enzyme activity was observed in the *amiR-bpm* line under control conditions. This suggests that proline may have contributed significantly to ROS detoxification. Indeed, proline has been shown to act as an ROS scavenger and plays a key role in maintaining cellular redox balance [54,55,56].

### 3.3. Reduced BPMs Expression Prolongs Elevated HSP Levels During Recovery from Heat Stress

Since heat stress is known to induce the expression of HSPs, the focus in this part of the study was on analysing HSP90 and HSP70 protein levels. As expected, all heat-treated seedlings showed a significant increase in HSP90 expression compared to the respective controls. This observation is consistent with previous studies that described a pronounced induction of molecular chaperones, particularly HSP90, in response to elevated temperatures [64,65,66]. Interestingly, HSP90 expression remained elevated even after a 24 h recovery period. This result is in agreement with the study by Charng et al. [67], which showed that HSP90 levels in *A. thaliana* seedlings peaked approximately 3 h after exposure to 37 °C, then gradually decreased and returned to control levels after a recovery period of 72 h. These results suggest that, despite the moderate and transient nature of the stress applied in our study, the effects on the cellular proteome may persist beyond the initial stress event. Elevated HSP90 levels during the recovery phase may play a crucial role in cellular repair by supporting the refolding or stabilisation of denatured proteins that are damaged during the heat episode [68]. Although HSP70 proteins are constitutively expressed in most cells and are highly upregulated in response to stress conditions, including heat, some members of the HSP70 family exhibit a rapid and transient induction pattern. These are referred to as “hit-and-run” chaperones due to their rapid activation and short-lived expression during stress responses [69,70]. This behaviour was also observed in the present study, in which HSP70 levels increased immediately after heat stress, but returned to baseline levels after 24 h in heat-treated *oeBPM1* and wild-type seedlings. Remarkably, HSP70 and HSP90 levels remained comparatively higher in heat-treated *amiR-bpm* seedlings than in *oeBPM1* and wild-type seedlings after recovery. The prolonged induction of HSPs may be attributed to differences in upstream regulatory pathways, possibly reflecting the increased expression of *HSFA3* observed in *amiR-bpm* seedlings. Since HSFA3 is a key transcriptional regulator for heat-responsive genes and is activated by DREB2A, its increased expression in *amiR-bpm* likely contributed to the prolonged induction of HSPs expression following heat stress. Indeed, transgenic tobacco plants (*Nicotiana tabacum*) overexpressing *DREB2A* show higher expression of downstream genes such as *HSP70-3* and *Hsp18p* when exposed to salt and osmotic stress compared to wild-type plants [71].

In this study, expression of *BPM2* increased in *amiR-bpm* and wild-type seedlings immediately after heat stress, which is consistent with the findings of Morimoto et al. [12], who identified *BPM2* as the major inducible *BPM* gene in response to elevated temperatures. No such induction was observed in *oeBPM1*, where *BPM1* was constitutively overexpressed. Considering that BPM1 and BPM2 proteins are closely related [72], it is plausible that high levels of *BPM1* in *oeBPM1* seedlings functionally suppressed *BPM2* induction. Morimoto et al. [12] also showed that all BPMs can interact with DREB2A, although BPM2 appears to play the predominant role. This suggests that increased *BPM1* levels in the *oeBPM1* line could compensate for *BPM2* and enhance DREB2A degradation, thereby reducing *HSFA3* and HSPs expression. In addition, Škiljaica et al. [11] have shown that the BPM1 protein accumulates at 37 °C. This observation supports the idea that BPM1 protein remained elevated in the *oeBPM1* line under heat stress and attenuated the DREB2A to HSFA3 cascade. Since BPM proteins mediate the degradation of DREB2A, the higher BPM1 levels in *oeBPM1*—accompanied by lower levels in *amiR-bpm* [6]—could explain the decreased *DREB2A* and *HSFA3* expression in *oeBPM1* and their enhancement in *amiR-bpm*. Notably, only wild-type seedlings showed upregulation of several native *BPM* genes during the recovery phase, possibly to buffer excessive DREB2A activity and maintain the balance between growth and response to stress [12]. The absence of such a compensatory response in both transgenic lines suggests that their regulatory capacity is unbalanced—overexpression of *BPM1* in *oeBPM1* might override the plasticity of the native BPM network, while in *amiR-bpm* reduced *BPM*s expression restricts overall regulation following stress and possibly prolongs DREB2A signalling.

## 4. Materials and Methods

### 4.1. Plant Material, Growth Conditions, and Treatment

This study was carried out on the model plant *Arabidopsis thaliana* (L.) Heynh., ecotype Columbia-0 (Col-0). The experimental setup included the wild type and two transgenic lines with altered *BPM*s gene expression: (1) the *oeBPM1* line, which overexpresses the *BPM1* gene and was generated by introducing the *BPM1*-*GFP* transgene under the control of the CaMV 35S promoter, allowing constitutive expression [11]; (2) the *amiR-bpm* line, in which the expression of *BPM1*, *BPM4*, *BPM5*, and *BPM6* was reduced by artificial microRNA technology [6].

For each line, approximately 3.5 mg of seeds were weighed into individual microtubes, with a total of 20 microtubes prepared per line. One microtube represented one biological replicate. Seeds were surface sterilised according to the protocol described by Vuković et al. [43] and transferred to sterile Petri dishes (6 cm diameter) containing 1 mL of liquid Murashige and Skoog (MS) medium [73] supplemented with vitamins and minerals (M5519, Sigma-Aldrich, Saint Louis, MO, USA), 1% (*w*/*v*) sucrose and 2.5 mM 2-(N-morpholino)ethanesulfonic acid (MES), adjusted to pH 5.7. The seeds were then stratified at 4 °C for three days before being transferred to a growth chamber to germinate and grow at 24 ± 1 °C, with a light intensity of 120–130 μmol m^−2^ s^−1^ and long-day conditions (16 h light/8 h dark). In the following 12 days, 1 mL of fresh MS medium, as described above, was added twice to each Petri dish under sterile conditions. On day 12, when the seedlings had two rosette leaves and reached developmental stage 1.02 [74], each of the three plant lines was divided into two experimental groups: (1) the control group, which was kept in the growth chamber at 24 °C; (2) the heat-treated group, which was placed in a preheated incubator (Hood TH 30, Edmund Bühler, Germany) at 37 °C. After 6 h of incubation, samples of the heat-treated seedlings (HS) were taken from half of the Petri dishes for physiological and biochemical analyses. Sampling immediately after 6 h of heat treatment represented the first time point. The remaining Petri dishes were returned to the growth chamber for a 24 h recovery period at 24 °C. The seedlings were collected after 24 h and labelled as the REC group, which represented the second time point. The control groups—C-HS and C-REC—were sampled at the same time points as HS and REC, respectively. For the molecular analysis, i.e., the analysis of gene expression, the seedlings were sampled at three time points—before treatment (C), immediately after treatment (HS), and after a recovery period of 24 h (REC). Prior to analysis, all seedlings were rinsed with distilled water to remove the MS medium.

### 4.2. Determination of Photosynthetic Efficiency

Photosynthetic efficiency and energy fluxes in PSII were evaluated using the JIP-test, which measures polyphasic Chl *a* fluorescence in dark-adapted plants [35]. Fluorescence induction and measurement was performed using a FluorPen FP 100 fluorometer (Photon Systems Instruments, Drásov, Czech Republic). For each measurement, approximately 50 seedlings per line and experimental group were pooled, representing one biological replicate. The seedlings were kept in the dark at 24 °C for 30 min on a tray covered with several layers of moist filter paper to prevent the seedlings from drying out. The polyphasic fluorescence increase was triggered by a blue light pulse (peak emission at 455 nm, photon flux density of 3000 μmol m^−2^ s^−1^). The FluorPen FP 100 recorded Chl *a* fluorescence intensity at 50 μs (F_0_), 2 ms (F_J_), 30 ms (F_I_) and after reaching the maximum fluorescence (F_M_). Fluorescence parameters describing the functional properties of PSII were read and analysed with the FluorPen 1.1 software: maximum quantum yield of PSII photochemistry (F_V_/F_M_), performance index on absorption basis (PI_ABS_), absorption flux per active reaction centre (ABS/RC), trapped energy flux per reaction centre (TR_0_/RC), electron transport flux per reaction centre (ET_0_/RC), and dissipated energy flux per reaction centre (DI_0_/RC). All results were expressed in arbitrary units (a.u.).

### 4.3. Measurement of Photosynthetic Pigment Content

Contents of Chl *a*, Chl *b*, and total Cars were determined according to the method of Wellburn [75]. For pigment extraction, approximately 50 mg of fresh plant tissue was homogenised in an ice-cold mortar and pestle with the addition of 1 mL of chilled 80% (*v*/*v*) acetone and approximately 20 mg of calcium carbonate. The homogenised mixture was centrifuged (5000× *g*, 10 min, 4 °C) and the supernatant collected, while the pellet was resuspended in 500 μL of chilled acetone, mixed, and centrifuged again under the same conditions. The supernatants were combined, and the final volume of each sample was adjusted to 1.5 mL with chilled acetone. Absorbance was measured at 470, 646, and 663 nm, and 80% (*v*/*v*) acetone was used as a blank. Pigment concentrations were calculated according to Wellburn [75], and the results were expressed as a portion of pigments in fresh tissue weight (μg g^−1^ FW).

### 4.4. Measurement of Hydrogen Peroxide, Malondialdehyde, and Proline Content

The hydrogen peroxide (H_2_O_2_) content was measured using the ferrous oxidation-xylenol orange (FOX) assay according to the method described by Mátai and Hideg [76]. The method is based on the oxidation of iron(II) ions (Fe^2+^) to iron(III) ions (Fe^3+^) by H_2_O_2_ under acidic conditions, whereby Fe^3+^ forms a coloured complex with xylenol orange, which is quantified spectrophotometrically. To prepare the plant extract, approximately 50 mg of fresh tissue was homogenised in an ice-cold mortar and pestle with 500 μL of chilled 70% (*v*/*v*) ethanol. The homogenised tissue was centrifuged (15,000× *g*, 10 min, 4 °C) and the supernatant was used for the determination of H_2_O_2_. The FOX reagent contained 124 μM xylenol orange, 99 mM sorbitol, and 0.248 mM ammonium iron(II) sulphate hexahydrate prepared in 2.5 M sulphuric acid. For each measurement, 1 mL of the FOX reagent was combined with 50 μL of the plant extract, briefly mixed, and incubated for 15 min at room temperature. The absorbance was measured at 560 nm against a blank sample prepared with 50 μL 70% (*v*/*v*) ethanol. The H_2_O_2_ concentration was calculated using a standard curve constructed from known H_2_O_2_ concentrations (1.82–72.8 μmol L^−1^) and expressed as nmol g^−1^ FW.

Lipid peroxidation was determined by measuring the level of malondialdehyde (MDA), a marker of membrane lipid damage, using the thiobarbituric acid (TBA) assay [77]. Approximately 200 mg of fresh tissue was homogenised in 2 mL of ice-cold 0.1% (*w*/*v*) trichloroacetic acid (TCA) with 20 mg polyvinylpolypyrrolidone (PVPP). The homogenate was centrifuged (15,000× *g*, 10 min, 4 °C) and the supernatant was collected. An aliquot of this extract was also used for the quantification of proline. For MDA quantification, 750 μL of 0.5% (*w*/*v*) TBA in 20% (*w*/*v*) TCA was added to 250 μL of each sample, followed by incubation at 100 °C for 30 min. After rapid cooling on ice, the samples were centrifuged (15,000× *g*, 5 min, 4 °C) and the supernatant was used to measure absorbance at 440, 532, and 600 nm. To correct for flavonoid interferences, absorbance at 532 and 600 nm was also measured in samples prepared with 750 μL of 20% (*w*/*v*) TCA without TBA and which underwent the same processing steps as the TBA-treated samples. Blanks were prepared with 250 μL 0.1% (*w*/*v*) TCA. The MDA content was calculated according to Hodges et al. [77] and expressed as nmol g^−1^ FW.

The proline content was determined using the ninhydrin reaction method described by Bates et al. [78] with slight modifications. As previously mentioned, the extract prepared for the determination of MDA was used. In brief, 400 μL of the extract was mixed with an equal volume (400 μL) of glacial acetic acid and 400 μL of acidic ninhydrin solution (0.14 M ninhydrin dissolved in a mixture of glacial acetic acid and 6 M phosphoric acid in a volume ratio of 1.5:1). The reaction mixture was thoroughly mixed and incubated for 1 h at 100 °C. After incubation, the mixture was cooled for 5 min. The reaction product was then extracted with 1 mL of toluene and the absorbance was measured at 520 nm with toluene as blank. The proline concentration was quantified using a standard curve constructed from known proline concentrations (1–500 μM) and expressed as µmol g^−1^ FW.

### 4.5. Protein Extraction and Antioxidant Enzyme Activity Assays

Total soluble proteins were extracted by homogenising approximately 150 mg of fresh seedlings with 15 mg of PVPP in an extraction buffer containing 100 mM potassium phosphate buffer (pH 7.0) and 0.1 mM ethylenediaminetetraacetic acid (EDTA). The homogenate was then centrifuged (20,000× *g*, 30 min, 4 °C) and the resulting supernatant was used for enzyme activity assays. Since total soluble protein extracts were used, the measured activities represent the combined (total) activity of all isoforms present in the seedlings without distinguishing between subcellular localisations. The protein concentration was determined using the Bradford assay [79] and calculated from a standard curve generated with bovine serum albumin (BSA) in a concentration range of 0.1–0.8 mg mL^−1^.

The activity of guaiacol peroxidase (G-POD, EC 1.11.1.7) was determined according to Maehly and Chance [80]. The reaction mixture (980 μL) consisted of 50 mM potassium phosphate buffer (pH 7.0), 18 mM guaiacol, and 4.5 mM H_2_O_2_. After addition of 20 μL of protein extract, the mixture was briefly mixed, and the absorbance was monitored at 470 nm every 15 s for 3 min. The enzyme activity was calculated using the extinction coefficient (26.6 mM cm^−1^) and expressed as μmol min^−1^ mg^−1^ proteins.

The activity of ascorbate peroxidase (APX, EC 1.11.1.11) was measured according to Nakano and Asada [81]. The reaction mixture consisted of 50 mM potassium phosphate buffer (pH 7.0) containing 0.1 mM EDTA, 20 mM ascorbate, and 12 mM H_2_O_2_, with a final volume of 1 mL, 180 μL of which was protein extract. After brief mixing, the absorbance was recorded at 290 nm every second for 15 s. Activity was determined using the extinction coefficient (2.8 mM cm^−1^) and expressed as μmol min^−1^ mg^−1^ proteins.

The catalase activity (CAT, EC 1.11.1.6) was determined as described by Aebi [82]. The reaction was started by adding 50 μL of protein extract to a mixture (950 μL) containing 50 mM potassium phosphate buffer (pH 7.0) and 10 mM H_2_O_2_. Enzyme activity was recorded as a decrease in absorbance at 240 nm every 10 s for 2 min, calculated using the extinction coefficient (40 mM cm^−1^) and expressed as nmol min^−1^ mg^−1^ proteins.

The activity of superoxide dismutase (SOD, EC 1.15.1.1) was determined according to the method of Beauchamp and Fridovich [83]. The reaction mixture consisted of 825 μL of SOD buffer (50 mM potassium phosphate buffer, pH 7.8, containing 0.1 mM EDTA, and 75 μM nitro blue tetrazolium chloride), 75 μL of 10.8 mM xanthine, 50 μL of 0.05 U mL^−1^ xanthine oxidase, 45 μL of protein extraction buffer, and 5 μL of protein extract. Absorbance was monitored at 560 nm every 30 s for 3 min. SOD activity was determined using a calibration curve (0.005–1 U μL^−1^) and expressed as U mg^−1^ proteins.

### 4.6. Immunodetection of Heat Shock Protein 70 and Heat Shock Protein 90

Soluble proteins were extracted from 50 mg of fresh seedlings with a Tris-HCl buffer (pH 8.0) containing 0.1 M Tris, 0.5 M sucrose, 6.5 mM dithiothreitol, and 8.25 mM cysteine–HCl. The samples were homogenised with 5 mg of PVPP and centrifuged (20,000× *g*, 30 min, 4 °C). The protein concentration was measured using the Bradford assay [79]. To denature the proteins, Laemmli sample buffer containing 87.5 mM Tris-HCl (pH 6.8), 2% (*w*/*v*) sodium dodecyl sulphate (SDS), 45% (*v*/*v*) glycerol, 12.5% (*v*/*v*) 2-mercaptoethanol, and 0.0125% (*w*/*v*) bromophenol blue was added [84], and the samples were heated at 95 °C for 5 min. For each sample, a volume containing 4 μg of proteins was calculated and loaded onto a gel. The denatured samples were separated by sodium dodecyl sulphate–polyacrylamide gel electrophoresis (SDS-PAGE) in a mini-vertical gel system (Mini-PROTEAN 3 Cell, BioRad, Hercules, CA, USA). Two acrylamide/bis-acrylamide gels with different pH values were used: a 4% stacking gel (pH 6.8) and a 12% resolving gel (pH 8.8). The running buffer (pH 8.3) contained 25 mM Tris, 192 mM glycine, and 0,1% (*w*/*v*) SDS. The separated proteins were transferred to a 0.45 µm nitrocellulose membrane at 60 V for 1 h using a wet transfer system (Mini Trans-Blot cell, BioRad). The transfer buffer (pH 8.3) consisted of 28 mM Tris, 192 mM glycine, and 10% (*v*/*v*) methanol. The efficiency of the transfer and equal loading of the samples were confirmed by staining the membrane with 0.05% (*w*/*v*) Ponceau S prepared in 5% (*v*/*v*) acetic acid, as described by Romero-Calvo et al. [85]. The dye was washed off by rinsing the membranes several times in dH_2_O. After blocking in 5% (*w*/*v*) non-fat milk in 1× Tris-buffered saline with 1% (*v*/*v*) Tween^®^ 20 (TBS-T) for 1 h at room temperature, the membranes were incubated overnight at 4 °C with primary antibodies against HSP70 (AS08371, Agrisera, Vännäs, Sweden) and HSP90 (AS08346, Agrisera), each diluted 1:3000 in blocking solution. After washing twice for 5 min in 1× TBS-T, the membranes were incubated with horseradish peroxidase (HRP)-conjugated secondary antibodies (goat anti-rabbit IgG, Merck Millipore, Burlington, MA, USA), diluted 1:10,000 in blocking solution, for 1 h at room temperature. Protein detection was performed using a chemiluminescent substrate (Immobilon Forte Western HRP, Merck Millipore) and blots were visualised using the C-DiGit Blot Scanner (LI-COR Biosciences, Lincoln, NE, USA). Band intensities were quantified using Image Studio™ Lite 5.2 software (LI-COR Biosciences).

### 4.7. Quantification of DREB2A, HSFA3 and BPMs Expression

Total RNA was extracted from approximately 20 mg of whole *A*. *thaliana* seedlings using the MagMAX™ Plant RNA Isolation Kit (Thermo Fisher Scientific, Waltham, MA, USA). The concentration and purity of RNA was determined using a NanoDrop™ 1000 spectrophotometer. A total of 1 µg of the isolated RNA was used for cDNA synthesis in a 20 µL reaction mixture containing 200 U RevertAid H Minus Reverse Transcriptase (Thermo Fisher Scientific), 1× Reaction Buffer (Thermo Fisher Scientific), 20 U RiboLock RNase Inhibitor (Thermo Fisher Scientific), 1 mM dNTP mix (Sigma-Aldrich), and 2.5 µM Oligo(dT)_18_ Primer (Thermo Fisher Scientific). The reaction was incubated at 65 °C for 5 min, at 42 °C for 45 min, and at 70 °C for 15 min. The resulting cDNA was diluted to a final concentration of 10 ng μL^−1^. To assess the quality of the cDNA, a standard PCR reaction was performed using *ACT* non-isoform-specific primers (Table 3). PCR products were analysed by electrophoresis on a 2% (*w*/*v*) agarose gel in 1× Tris–acetate–EDTA buffer (1 mM EDTA in 40 mmol L^−1^ Tris–acetate, pH 8.0).

For quantitative PCR analysis (qPCR), the final reaction mixture (15 μL) contained 2 μL of cDNA, 1× GoTaq^®^ qPCR Master Mix (Promega, Madison, WI, USA) and 200 nM gene-specific primers (Table 3). Reactions were run on a Magnetic Induction Cycler (Mic qPCR, Bio Molecular Systems, Upper Coomera, QLD, Australia) under the following thermal cycling conditions: 1 cycle at 95 °C for 5 min, 40 cycles at 95 °C for 5 s, followed by 1 cycle at 60 °C for 10 s. The specificity of the amplification was confirmed by melting curve analysis in which the temperature increased from 50 °C to 95 °C at a rate of 0.5 °C per s. The internal control genes *OGIO* and *PUX7* (Table 3) were used for normalisation [86]. Relative gene expression levels were calculated using the ΔΔCt method [87,88] and normalised to the wild-type control seedlings sampled before treatment (wt C). For the *BPM1*-*GFP* transgene, expression was also normalised, but to the endogenous *BPM1* gene in the control *oeBPM1* seedlings sampled before treatment (*oeBPM1* C).

**Table 3:** Primers used for the amplification of the genes of interest in standard (*) and quantitative real-time PCR. Primer efficiencies were calculated using the PCR analysis software 2.12.7 of the real-time PCR instrument (Mic qPCR Analysis Software, Bio Molecular Systems).

Gene	Accession Number	5′ → 3′ Sequence (Forward/Reverse)	Primer Efficiency	Reference
*ACT* *	At3g53750	CTGGCATCATACTTTCTACAATG CACCACTGAGCACAATGTTAC	/	[43]
*DREB2A*	At5g05410	CAGTGTTGCCAACGGTTCAT AAACGGAGGTATTCCGTAGTTGAG	0.87	[86]
*HSFA3*	At5g03720	AGTTTGCCAGAATCATACTTCCA AGCAAGTTTGGTTGGATTGTGG	0.82	[11]
*BPM1*	At5g19000	CCCGGTTGCACTGAATGGGA ACGATTCATTGTACTTGCTAGATCCGATT	0.90	[11]
*BPM2*	At3g06190	TCTATCCGGGTAATAAGATCGAAGA CCTTGGAAACCCTAATTGTGTC	0.86	[11]
*BPM3*	At2g39760	AGTGATAGACGACATCGAACCT CAAGGTCATAGAGGTCAGCA	0.86	[11]
*BPM4*	At3g03740	GAAGTTACTGACATGGAGCCT CACTGACTCGCACATTAGAC	0.84	[11]
*BPM5*	At5g21010	CGTTTGCCTTAAGTTTACTGCC ACTGTTACTACCTTCCTCGTG	0.78	[89]
*BPM6*	At3g43700	AAGGGTCAGGCAGCGAACCA CCGCTTCCCTTTCATTCGGTACA	0.94	[89]
*BPM1-GFP*	/	AGTGGAAGACGAGTGAAGC CTGAACTTGTGGCCGTTTAC	0.85	[89]
*OGIO*	At5g51880	ATCCAAGAGCAGTTCAAGCAAG GAGAGCCATACCTTCCACTG	0.82	[86]
*PUX7*	At1g14570	GTTTCTCAGACTATCAAAGCCA ATCAATTACAAGCACCACGG	0.86	[86]

Abbreviations: *ACT*—gene family encoding actin isoforms; *BPM1*-*6*—gene family encoding MATH-BTB proteins in *Arabidopsis thaliana*; *DREB2A*—gene encoding dehydration-responsive element-binding protein 2A; *HSFA3*—gene encoding heat stress transcription factor A-3; *OGIO*—gene encoding 2-oxoglutarate (2OG) and Fe(II)-dependent oxygenase superfamily protein; *PUX7*—gene encoding plant UBX-domain-containing protein.

### 4.8. Statistical Analysis

Each experiment was performed independently three times, and one representative result was presented. Before performing statistical analyses, the data were checked for outliers using Tukey’s fences, with k set to 1.5. The distribution of variables was assessed using the Shapiro–Wilk W test, while variance homogeneity was assessed with Levene’s test. Data were considered normally distributed, and variances were considered equal if *p* > 0.05. As the data followed a normal distribution and showed equal variances, parametric statistical analyses were applied.

A two-way ANOVA was performed to evaluate the effects of the heat treatment, the tested lines, and their interactions on the measured parameters. This analysis was used to determine whether the observed changes were significantly influenced by the treatment, the tested lines, or the combination of both factors. If the two-way ANOVA revealed a statistically significant effect, we conducted further analyses to determine specific group differences. For comparisons involving only two groups (C-HS vs. HS, or C-REC vs. REC within wild type, *oeBPM1*, or *amiR-bpm*), Student’s *t*-test for independent samples was used. For comparisons involving plant lines (wild type vs. *oeBPM1* vs *amiR-bpm*) exposed to the same treatment condition (C-HS, HS, C-REC, or REC), a one-way ANOVA followed by Tukey’s HSD *post*-*hoc* test was performed.

Gene expression was analysed in seedling tissue sampled at three different time points (before heat exposure, immediately after exposure and after a 24 h recovery period). Therefore, a two-way repeated measures ANOVA was performed. For the analysis, the tested lines were defined as fixed factors and the time points as repeated factors. To assess changes in gene expression within each line, paired Student’s *t*-test was performed comparing the C and HS groups or the C and REC groups. For comparisons involving the same treatment condition (C, HS, or REC) between wild type, *oeBPM1*, and *amiR-bpm*, a one-way ANOVA followed by Tukey’s HSD *post*-*hoc* test was performed.

All differences were considered significant at *p* ≤ 0.05. Statistical analyses were performed with Statistica 14.0 (TIBCO Software Inc., Palo Alto, CA, USA).

## 5. Conclusions

This study provides new insights into the role of BPM proteins in modulating the response of *A. thaliana* to moderate heat stress. By analysing *A. thaliana* lines with altered expression of *BPM* genes, we demonstrated that BPM proteins influence important physiological and molecular processes, including photosynthesis, antioxidant activity, the accumulation of HSPs, and the expression of heat-responsive genes. The results suggest that overexpression of *BPM1* can disrupt the balance between stress signalling and cellular homeostasis, while reduced expression of several *BPM* genes, as in the *amiR-bpm* line, may enable a more sustained yet regulated activation of heat-responsive pathways such as DREB2A to HSFA3 cascade, promoting a more adaptive response to moderate heat stress. The response to heat stress was also associated with line-specific changes in the expression of *BPM* genes, supporting the regulatory interplay between BPM proteins and heat signalling components. Our results emphasise the importance of balanced expression of *BPM*s in coordinating stress responses and suggest that BPM1 and possibly other BPM proteins contribute to different physiological strategies. In addition, this study provides a basis for future research on the stress response regulated by BPM proteins, their involvement in proteostasis, and their potential application to improve heat stress tolerance in plants.

## Figures and Tables

**Table 1:** Specific energy fluxes—absorption (ABS/RC), trapping (TR_0_/RC), electron transport (ET_0_/RC), and dissipation of energy as heat (DI_0_/RC) per active PSII reaction centre (RC)—in *Arabidopsis thaliana* seedlings with *BPM1* overexpression (*oeBPM1*), seedlings with *BPM1*, *4*, *5*, and *6* downregulation (*amiR-bpm*), and wild-type (wt) seedlings. Measurements were taken at two time points: immediately after exposure to 37 °C for 6 h (HS) and after a 24 h recovery at 24 °C (REC). The control groups (C-HS and C-REC) were kept at 24 °C throughout the experiment. Data are expressed as mean ± standard error (n = 5 biological replicates). Differences between two groups—control and heat-treated seedlings sampled at the same time point (immediately after the treatment or after 24 h recovery, i.e., C-HS vs. HS, or C-REC vs. REC)—were determined using Student’s *t*-test. Significant differences (*p* ≤ 0.05) between C-HS and HS are marked with an asterisk (*). No significant differences were found between C-REC and REC. For comparisons of the tested lines (wild type vs. *oeBPM1* vs. *amiR-bpm*) exposed to the same treatment condition (C-HS, HS, C-REC, or REC), a one-way ANOVA followed by Tukey’s HSD post hoc test was performed, with significant differences (*p* ≤ 0.05) indicated by different uppercase letters.

LINE	GROUP	ABS/RC	TR_0_/RC	ET_0_/RC	DI_0_/RC
wt	C-HS	3.02 ± 0.03 A	2.32 ± 0.02 A	1.47 ± 0.02 A	0.70 ± 0.02 A
	HS	3.00 ± 0.14 A	2.17 ± 0.06 A *	1.28 ± 0.04 A *	0.83 ± 0.09 A
	C-REC	2.94 ± 0.10 A	2.18 ± 0.06 A	1.29 ± 0.06 A	0.76 ± 0.05 A
	REC	2.91 ± 0.08 A	2.20 ± 0.05 A	1.30 ± 0.08 A	0.71 ± 0.03 A
*oeBPM1*	C-HS	2.69 ± 0.09 B	2.08 ± 0.07 B	1.26 ± 0.05 B	0.62 ± 0.02 B
	HS	3.11 ± 0.08 A *	2.25 ± 0.04 A	1.37 ± 0.04 A	0.86 ± 0.05 A *
	C-REC	2.70 ± 0.08 A	2.06 ± 0.05 A	1.28 ± 0.04 A	0.65 ± 0.04 A
	REC	2.62 ± 0.03 B	2.04 ± 0.03 A	1.29 ± 0.02 A	0.58 ± 0.01 B
*amiR-bpm*	C-HS	2.73 ± 0.04 B	2.14 ± 0.02 B	1.30 ± 0.02 B	0.59 ± 0.02 B
	HS	2.80 ± 0.03 A	2.14 ± 0.02 A	1.33 ± 0.01 A	0.66 ± 0.02 A *
	C-REC	2.74 ± 0.01 A	2.09 ± 0.03 A	1.28 ± 0.04 A	0.64 ± 0.02 A
	REC	2.70 ± 0.08 AB	2.09 ± 0.07 A	1.29 ± 0.05 A	0.61 ± 0.03 B

**Table 2:** Chlorophyll *a* (Chl *a*), chlorophyll *b* (Chl *b*), and total carotenoids (Cars) content in *Arabidopsis thaliana* seedlings with *BPM1* overexpression (*oeBPM1*), seedlings with *BPM1*, *4*, *5*, and *6* downregulation (*amiR-bpm*), and wild-type (wt) seedlings. Samples were collected at two time points: immediately after exposure to 37 °C for 6 h (HS) and after a 24 h recovery at 24 °C (REC). The control groups (C-HS and C-REC) were kept at 24 °C throughout the experiment. Data are expressed as mean ± standard error (n = 5 biological replicates). Differences between two groups—control and heat-treated seedlings sampled at the same time point (immediately after the treatment or after 24 h recovery, i.e., C-HS vs. HS, or C-REC vs. REC)—were determined using Student’s *t*-test. Significant differences (*p* ≤ 0.05) between C-HS and HS are marked with an asterisk (*). No significant differences were found between C-REC and REC. For comparisons of the tested lines (wild type vs. *oeBPM1* vs. *amiR-bpm*) exposed to the same treatment condition (C-HS, HS, C-REC, or REC), a one-way ANOVA followed by Tukey’s HSD post hoc test was performed, with significant differences (*p* ≤ 0.05) indicated by different uppercase letters.

LINE	GROUP	Chl *a* (μg g^−1^ FW)	Chl *b* (μg g^−1^ FW)	Cars (μg g^−1^ FW)
wt	C-HS	356.45 ± 20.80 AB	84.41 ± 3.52 A	105.07 ± 3.40 AB
	HS	313.18 ± 17.68 A	76.86 ± 1.93 A	86.84 ± 4.42 A *
	C-REC	305.22 ± 32.29 A	82.03 ± 11.51 A	88.08 ± 9.17 A
	REC	333.02 ± 16.18 A	92.31 ± 1.09 A	95.27 ± 4.06 A
*oeBPM1*	C-HS	405.41 ± 21.87 A	99.92 ± 6.58 A	116.08 ± 4.64 A
	HS	331.89 ± 12.99 A *	84.97 ± 3.68 A	100.77 ± 3.97 A *
	C-REC	357.97 ± 24.00 A	89.37 ± 5.99 A	104.69 ± 6.77 A
	REC	376.84 ± 25.17 A	98.78 ± 7.28 A	108.63 ± 5.95 A
*amiR-bpm*	C-HS	314.12 ± 22.23 B	82.97 ± 4.53 A	90.84 ± 5.94 B
	HS	368.84 ± 34.23 A	86.87 ± 6.98 A	104.22 ± 9.82 A
	C-REC	326.65 ± 9.45 A	83.57 ± 6.10 A	97.56 ± 0.62 A
	REC	328.29 ± 25.87 A	83.95 ± 6.50 A	97.00 ± 8.25 A

## Data Availability

Data are included in the article.
